# High Expression of Somatostatin Receptors 2A, 3, and 5 in Corticotroph Pituitary Adenoma

**DOI:** 10.1155/2018/1763735

**Published:** 2018-12-09

**Authors:** Felix Behling, Jürgen Honegger, Marco Skardelly, Irina Gepfner-Tuma, Ghazaleh Tabatabai, Marcos Tatagiba, Jens Schittenhelm

**Affiliations:** ^1^Department of Neurosurgery, University Hospital Tuebingen, Eberhard-Karls-University Tuebingen, Germany; ^2^Center for CNS Tumors, Comprehensive Cancer Center Tuebingen-Stuttgart, University Hospital Tuebingen, Eberhard-Karls-University Tuebingen, Germany; ^3^Department of Neurology, University Hospital Tuebingen, Eberhard-Karls-University Tuebingen, Germany; ^4^Hertie Institute for Clinical Brain Research, Tuebingen, Germany; ^5^Interdisciplinary Division of Neuro-Oncology, University Hospital Tuebingen, Eberhard-Karls-University Tuebingen, Germany; ^6^German Cancer Consortium (DKTK), DKFZ partner site Tuebingen, Germany; ^7^Department of Neuropathology, University Hospital Tuebingen, Eberhard-Karls-University Tuebingen, Germany

## Abstract

The development of somatostatin analogs for the treatment of pituitary Cushing's disease has been based on somatostatin receptor expression analyses of small cohorts of pituitary adenomas. Additionally, the classification of pituitary adenomas has recently changed. To enable progress with this treatment option, we assessed somatostatin receptors in a large cohort of corticotroph and other pituitary adenomas according to the new WHO classification of endocrine tumors. Paraffin-embedded tumor samples of 88 corticotroph pituitary adenomas and 30 nonadenomatous pituitary biopsies were analyzed after processing into tissue microarrays and immunohistochemical staining for SSTR 1, SSTR2A, SSTR3, SSTR4, and SSTR5. For comparison, 159 other noncorticotroph pituitary adenomas were analyzed. SSTR3 expression was higher in corticotroph adenomas compared to PIT-1-positive, gonadotroph, and nonfunctioning pituitary adenomas (*p* < 0.0001, *p* = 0.0280, and *p* < 0.0001, respectively). This was also the case for the expression of SSTR5 (*p* = 0.0003, *p* < 0.0001, and *p* < 0.0001, respectively). SSTR2A expression was higher compared to gonadotroph and nonfunctioning pituitary adenomas (*p* = 0.0217 and 0.0126, respectively) while PIT-1-positive adenomas showed even higher SSTR2A expression (*p* < 0.0001). SSTR2A and SSTR5 were both expressed higher in nonadenomatous pituitary biopsies than in pituitary adenomas (*p* = 0.0126 and *p* = 0.0008, respectively). There are marked expression differences of SSTR1-5 as well as changes in expression in recurrent disease that need to be addressed when looking for other possible substances for the treatment of Cushing's disease. SSTR2A, SSTR3, and SSTR5 seem to be most suitable biomarkers for a targeted therapy with somatostatin analogs.

## 1. Introduction

Cushing's disease (CD) is caused by an overproduction of the adrenocorticotroph hormone (ACTH) from the adenomatous tissue of the pituitary gland leading to hypercotisolism and the typical clinical and morphological changes [1, 2]. Roughly 15% of all pituitary adenomas are corticotroph adenomas arising from TPIT-lineage adenohypophyseal cells (TPIT = T-box protein 19) with a peak incidence in patients of 30-50 years [3]. Approximately 20% of the corticotroph adenomas lack ACTH excess but these silent adenomas may cause neurological or ophthalmological symptoms as macroadenomas. Histologically, corticotroph adenomas are classified either as densely or sparsely granulated or as Crooke cell adenomas depending on their ACTH expression pattern [4].

Consequences of untreated chronic glucocorticoid excess are increased mortality, mainly caused by cardiovascular changes. If successful treatment leads to a remission of the hypercortisolism, the mortality rate may return to a normal level [5, 6].

The first line treatment is the complete surgical resection of the ACTH-producing pituitary adenoma which achieves normalization of cortisol levels in 76% with a rate of recurrence of 10% [1, 7]. These cases need to be addressed with further local treatments including repeat-resections, radiation therapy [7], or as a last resort, bilateral adrenalectomy [8, 9]. A medical treatment that achieves high rates of sustained normalization of hypercortisolemia without significant side effects has not yet been discovered [10].

Somatostatin is a centrally acting cyclic peptide that inhibits the effects of the growth hormone somatotropin [11]. It also blocks the secretion of ACTH [12, 13] from the pituitary gland via somatostatin receptors (SSTRs). Substances addressing several somatostatin receptor subtypes with high affinity have been developed and clinically assessed in Cushing's disease [14]. Boscaro et al. conducted a multicenter phase 2 trial in 2009, where pasireotide decreased UFC levels in 76% of patients suffering from Cushing's disease [15]. A phase 3 trial by Colao et al. followed in 2012, which showed a significant decrease in cortisol levels [16].

The choice to target somatostatin receptors is based on the analysis of expression levels of SSTRs in relatively small groups of human corticotroph pituitary adenomas ranging from 1 to 13 cases using RT-PCR [17–22] and the experience that had been gained with inhibitory substances in other endocrine tumors and diseases [21].

Somatostatin receptors can also be utilized as a target for peptide receptor radionuclide therapy (PRRT). This treatment option has been developed for advanced endocrine gastroenteropancreatic tumors like gastrinomas [23, 24] and is also applied in selected cases of recurrent meningioma [25]. There is no sufficient data regarding PRRT in CD but the response to standard radiation therapy of recurrent Cushing's disease has been reviewed recently and showed good efficacy after short-term follow-up [26].

In order to move forward with this promising target for medical and nuclear medical treatment, we conducted this study, in which we analyzed the immunohistochemical expression of SSTR1, 2A, 3, 4, and 5 in a large group of 277 human pituitary adenomas including 88 corticotroph pituitary adenomas using tissue microarrays, classified according to the latest changes introduced in the WHO 2017 classification of endocrine tumors. The methodology of this study allows a clear statement about the expression levels of all 5 somatostatin receptors in routine diagnostics with regard to differentiating real tumor cell expression from false-positive expression in vascular structures or other adjacent nonadenomatous tissue.

## 2. Material and Methods

### 2.1. Patient Cohort

An electronic database search revealed 164 patients who suffered from Cushing's disease and who were treated in the authors' department from October 2004 to July 2015. Paraffin-embedded tumor tissue samples of 148 cases of Cushing's disease were available for analysis. In thirty cases of CD, no adenoma could be identified in the obtained tissue available for neuropathological examination. After tissue microarrays were constructed and validated, 15 cases did not have sufficient tumor tissue left for microscopic evaluation, and 12 samples showed PIT-1 positivity resulting in reclassification of these samples according to the new WHO classification of 2017. Overall, 88 samples with ACTH-producing tumors were available for further analysis. For comparative analysis, the 30 nonadenomatous anterior pituitary samples were processed into TMAs as well, together with 55 PIT-1-positive, 22 gonadotroph, and 82 nonfunctioning pituitary adenomas. A few cases of each group were lost for final immunohistochemical assessment because not enough tissue was successfully transferred on glass slides for analysis after the TMA procedure.

### 2.2. Tissue Microarray and Immunohistochemistry

**Figure 1:**
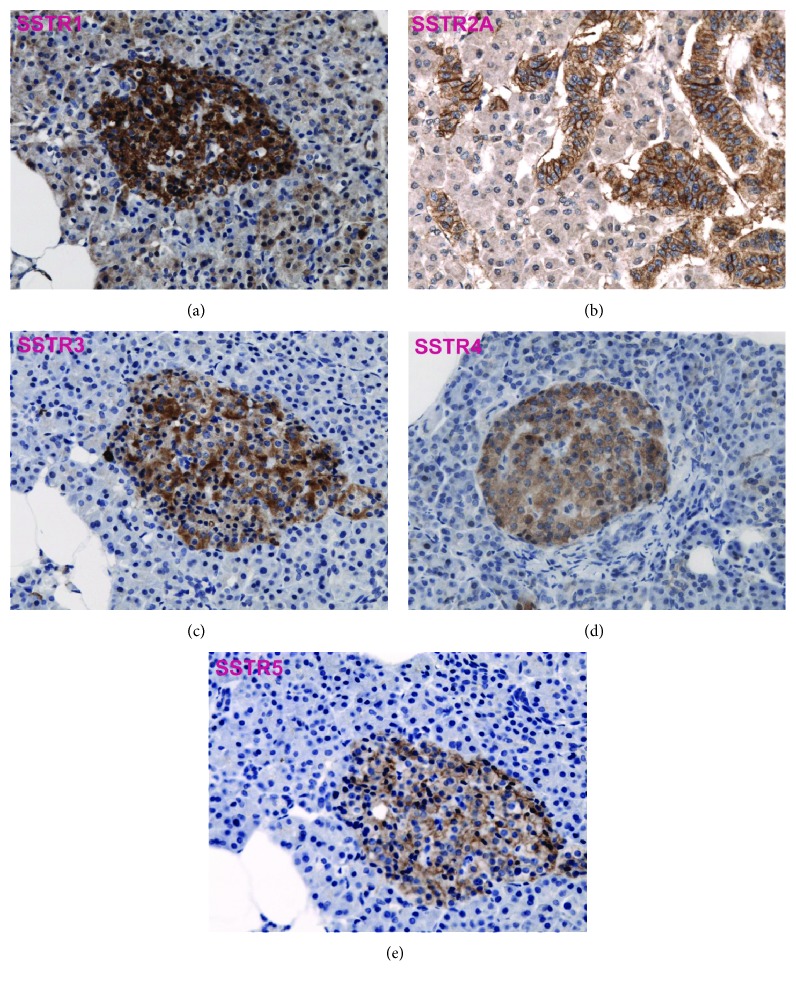
SSTR1, 2A, 3, 4, and 5 in pancreatic tissue show staining of the pancreatic islets which served as positive controls.

The histopathological reevaluation was done according to the latest WHO classification of tumors of the pituitary gland from 2017 [4]. Paraffin-embedded tumor tissue samples from the archive of the Institute of Neuropathology of the University Hospital Tübingen were retrieved. After microscopic evaluation of H&E stains, eligible tumor samples for cylinder extraction were marked on all paraffin-embedded tissue probes. With a conventional tissue microarrayer (Beecher Instruments, Sun Prairie, Wisconsin, USA), tumor cylinder probes 1 or 2 mm in diameter, depending on the available tumor tissue mass, were taken from the paraffin-embedded tumor probes and aligned on a recipient paraffin block in a chessboard pattern. In most cases, two sample cylinders were taken for each tumor. 4 *μ*m slices were produced from the TMA blocks and dried at 80°C for 15 minutes. Subsequent immunohistochemical staining was done with a Ventana BenchMark immunostainer (Ventana Medical Systems, Tucson, Arizona, USA). CC1 pretreatment was performed using the OptiView method for 8-72 min (except for SSTR3 with protease pretreatment for 4 min), followed by incubation with primary antibodies (SSTR1: 1 : 3000 (Gramsch, Schwabhausen, Germany), SSTR2A: 1 : 500 (Dianova, Hamburg, Germany), SSTR3: 1 : 250 (Abcam, Cambridge, United Kingdom), SSTR4: 1 : 1000 (Gentex, Zeeland, United States), SSTR5: 1 : 100 (Abcam, Cambridge, United Kingdom), alpha-subunit: 1 : 400 (Immunotech, Prague, Czech Republic), PIT-1: 1 : 200 (8 min) (Cell Signaling Technology, Danvers, United States)) at 37°C for 32 min if not stated otherwise. A cerebral metastasis of a neuroendocrine pancreatic tumor, a cerebral breast cancer metastasis, and normal pancreatic tissue were used as controls for specific SSTR expression (see Figure 1).

**Figure 2:**
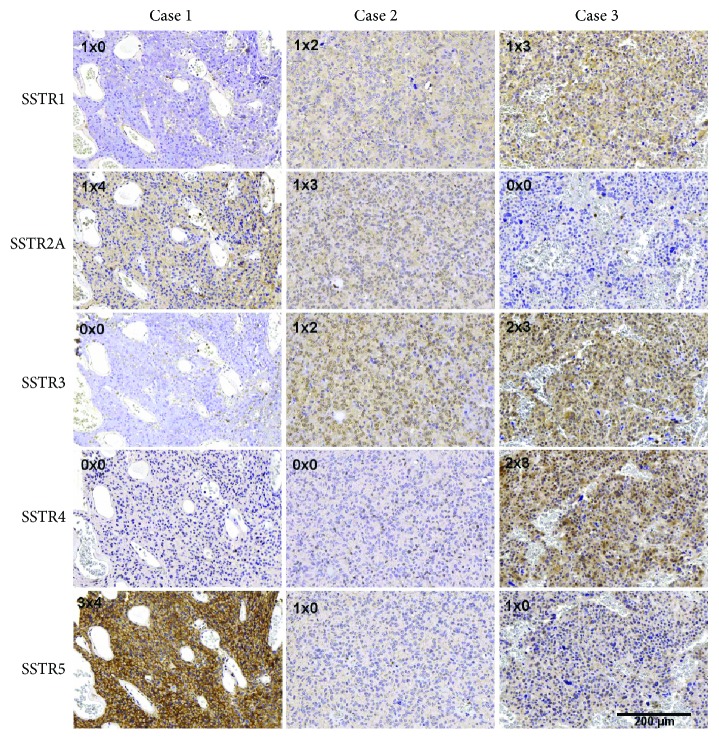
Scoring of the SSTR expression according to Barresi et al. 2008 [27]. The intensity distribution (ID) score = immunostaining intensity (IS) *×* area of staining positivity (ASP) and is shown in the left upper corner. The examples show the expression of SSTR1, 2A, 3, 4, and 5 in three cases of corticotroph pituitary adenoma, ranging from 0 to the maximum ID score of 12.

The stained TMA slides were microscopically evaluated regarding the presence of tumor tissue and expression of pituitary hormones, alpha-subunit, and PIT-1. Subsequently, the samples were subgrouped into corticotroph, PIT-1-positive, gonadotroph, and nonfunctioning pituitary adenomas as well as nonadenomatous tissue samples. Quantification of SSTR expression (cytoplasmic and membranous) was done according to the previously established scoring system by Barresi et al. [27]. Considering the immunostaining intensity (IS) and the area of staining positivity (ASP), an intensity distribution (ID) score was generated by the multiplication of IS and ASP, ranging from 0 to 12 (see Table 1 and Figure 2). We used an ID score of 6 as a cut-off to distinguish samples with high expression rates. A score below 1 was considered negative. The same scoring system and cut-off were used previously by Barresi et al. who described SSTR expression in meningiomas [27]. The number of samples available for statistical analysis varies between the different markers, due to the vertical heterogeneity of the tumor cylinders and inadequate fixation or staining of tumor tissue in a few single cases.

### 2.3. Study Design

This retrospective observational single-center study was designed to analyze the distribution and expression of somatostatin receptors (SSTRs) 1, 2A, 3, 4, and 5 in Cushing's disease and other pituitary adenomas (1) together with clinical aspects (2), such as age, gender, primary/recurrent disease, and hormone expression. The study was approved by the Clinical Ethics Committee of the University of Tübingen (Project number: 618/2014BO2) and complies with the Declaration of Helsinki.

### 2.4. Statistical Methods

For analysis of the ID score for the expression of SSTR1–5 as a continuous variable, ANOVA followed by a Student's *t*-test was applied with a significance level of *α* < 0.05. Comparison of the ID score cut-off at 6 was done by contingency tables with Pearson's chi square test, also with a significance level of *α* < 0.05. Statistical analysis was done with Microsoft® Excel® (Redmond, WA) and JMP® Version 13.1 (SAS; Cary, NJ).

### 2.5. Image Preparation

Stained slides were scanned using a Zeiss Mirax slide scanner (Zeiss; Göttingen, Germany); images were taken using the Mirax Viewer software (Zeiss; Göttingen, Germany) and figures created with GIMP (Version 2.8.22 by Spencer Kimball, Peter Mattis, and the GIMP Development Team).

## 3. Results

### 3.1. Demographic Characteristics

Overall, 247 pituitary adenomas were analyzed. In 88 cases, the patients suffered from Cushing's disease including 20 recurrent adenomas (23%). In thirty additional cases of CD, no adenoma was identified in the obtained pituitary tissue biopsies. The median age at the time of surgery was 49 years for patients with corticotroph adenomas, similar to the other analyzed adenoma subtypes. The distribution of age, gender, and tumor recurrence rate is outlined in detail in Table 2.

### 3.2. SSTR1

**Figure 3:**
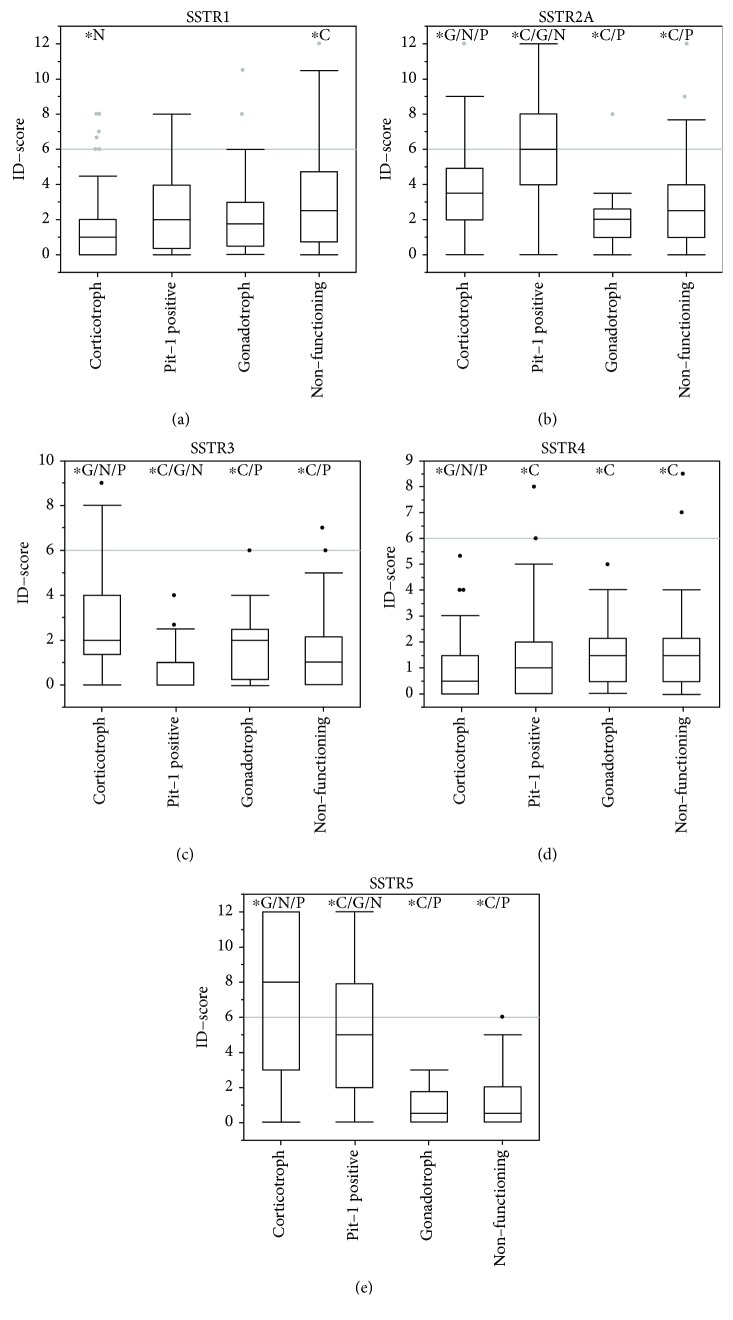
Expression of SSTR1 (a), SSTR2A (b), SSTR3 (c), SSTR4 (d), and SSTR5 (e) in pituitary adenomas. Asterisks mark significant differences compared to other pituitary adenomas (corticotroph (C), gonadotroph (g), PIT-1-positive (P), and nonfunctioning pituitary adenomas (N)) according to Student's *t*-test with a significance of *α* < 0.05. Outliers are represented as single points and were included in each analysis.

Tissue samples of 236 pituitary adenomas were available for microscopic evaluation of SSTR1 expression. The mean ID score for all adenoma samples was 2.47 (95% CI 2.15–2.78). Sixty-five samples were negative (28%, ID score < 1). Nonfunctioning adenomas had the highest mean ID score (3.07, 95% CI 2.46–3.68) followed by PIT-1-positive (2.53, 95% CI 1.90–3.17) and gonadotroph adenomas (2.52, 95% CI 1.32–3.72). Corticotroph adenomas showed the lowest mean ID score (1.79, 95% CI 1.34–2.25) which was significantly lower compared to nonfunctioning pituitary adenomas (*p* = 0.001, see Table 3 and Figure 3(a)). There was no significant difference within the other adenoma subgroups regarding the mean ID score. Overall, thirty-three samples were scored above the cut-off of 6 (14%, 33/236), while 203 were below (86%, 203/236). Inactive adenomas showed the highest rate of SSTR1 expression above the cut-off (20%, 16/81), followed by gonadotroph (14%, 3/22), PIT-1-positive (13%, 7/54), and corticotroph adenomas (9%, 7/79). However, the differences were not statistically significant (*p* = 0.2599, see Table 4).

### 3.3. SSTR2A

Immunohistochemical evaluation of 226 pituitary adenoma samples regarding SSTR2A expression was done. The mean ID score was 3.88 (95% CI 3.50–4.27) with a total of 92% immunopositive samples (209/226), while 17 samples were negative (8%, 17/226). PIT-1-positive adenomas had the highest mean ID scores with 6.43 (95% CI 5.50–7.37), followed by corticotroph (3.73, 95% CI 3.19–4.26), nonfunctioning (2.70, 95% CI 2.21–3.19), and gonadotroph adenomas (2.19, 95% CI 1.31–3.08, see Table 3 and Figure 3(b)). The differences between the adenoma subgroups were all significant except for the comparison of nonfunctioning and gonadotroph adenomas (*p* = 0.4462, see Figure 3(b)). The ID score cut-off of 6 was reached in 58 cases (26%, 58/226) and 168 samples were scored lower (74%, 168/226). High expression of SSTR2A was seen in 24% of CD (19/80), 63% of PIT-1-positive (33/52), 6% of gonadotroph (1/18), and 7% of nonfunctioning adenoma patients (5/76), as delineated in Table 4. The differences were highly significant (*p* < 0.0001).

### 3.4. SSTR3

Somatostatin receptor 3 expression was evaluated in 232 pituitary adenoma samples. Sixty-two percent were scored immunopositive (62%, 145/232) while 38% were negative (87/232). The mean ID score was 1.76 (95% CI 1.52–2.01). Corticotroph adenoma samples had the highest mean ID score with 2.91 (95% CI 2.42–3.39) while gonadotroph (1.98, 95% CI 1.23–2.73), nonfunctioning (1.42, 95% CI 1.06–1.78), and PIT-1-positive adenomas (0.51, 95% CI 0.27–0.75) had significantly lower mean ID scores (*p* < 0.0001, *p* < 0.0001, and *p* = 0.028, respectively). While the mean ID score for PIT-1-positive adenomas was significantly lower than gonadotroph and nonfunctioning adenomas (*p* = 0.001 and *p* = 0.0026, respectively), the difference between the latter two subtypes was not significant (*p* = 0.1908) as illustrated in Figure 3(c) and Table 3. Only 18 cases reached the ID score cut-off (8%) with the remaining 214 samples scoring below 6 (92%, 214/232). These eighteen samples consisted of 15 CD cases (19%, 15/79) and two nonfunctioning (3%, 2/78) and one gonadotroph adenoma (5%, 1/20). None of the 54 PIT-1-positive adenomas reached the ID score cut-off of 6 (see Table 4). Overall, the observed frequencies were significantly different (*p* = 0.0001).

### 3.5. SSTR4

A total of 235 adenoma samples were stained for SSTR4 expression. Sixty percent were immunopositive (60%, 142/235) while 40% did not express SSTR4 (39%, 93/235). Gonadotroph and nonfunctioning pituitary adenomas had the highest mean SSTR4 expression (both 1.64, 95% CI 1.02–2.25 and 1.31–1.98, respectively) followed by PIT-1-positive adenomas (1.44, 95% CI 0.97–1.91). With 0.90 (95% CI 0.64–1.15), corticotroph adenomas had a significantly lower mean ID score than gonadotroph, nonfunctioning, and PIT-1-positive adenomas (*p* = 0.0341, *p* = 0.0013, and *p* = 0.328, respectively). The differences between these three subgroups were not significant (see Table 3 and Figure 3(d)). Only 4 samples scored above the cut-off ID score (2%, 4/235); two nonfunctioning (3%, 2/78) and 2 PIT-1-positive adenomas (4%, 2/54). The remaining samples showed SSTR4 expression below the cut-off (231/235). All corticotroph (*n* = 81) and gonadotroph adenomas (*n* = 22) scored expression rates below the cut-off. Thus, statistical significance was not reached (*p* = 0.3309, see Table 4).

### 3.6. SSTR5

Overall, 233 adenoma cases were available for SSTR5 expression analysis. Seventy samples were immunonegative (30%, 70/233) while 163 showed positive staining (70%, 163/233). Corticotroph adenomas had the highest mean ID score (7.11, 95% CI 6.11-8.12), significantly higher than PIT-1-positive (4.96, 95% CI 3.96–5.97, *p* = 0.0003), nonfunctioning (1.16, 95% CI 0.83–1.49, *p* < 0.0001), and gonadotroph adenomas (0.90, 95% CI 0.50–1.31, *p* < 0.0001). The differences between PIT-1-positive and gonadotroph adenomas as well as nonfunctioning adenomas were also highly significant (each *p* < 0.0001), while no significant difference was found between nonfunctioning and gonadotroph adenomas (*p* = 0.7572, see Table 3 and Figure 3(e)). The ID score cut-off was reached in 32% (74/233) and 68% scored lower expression rates (159/233). High rates above the cut-off were observed for corticotroph (59%, 48/82) and PIT-1-positive adenomas (48%, 25/52). All gonadotroph adenomas (0/21) and all but one nonfunctioning adenoma (1/78) did not reach the ID score cut-off of 6 (see Table 4). The observed differences were statistically significant (*p* < 0.0001).

### 3.7. Recurrent Pituitary Adenomas

**Figure 4:**
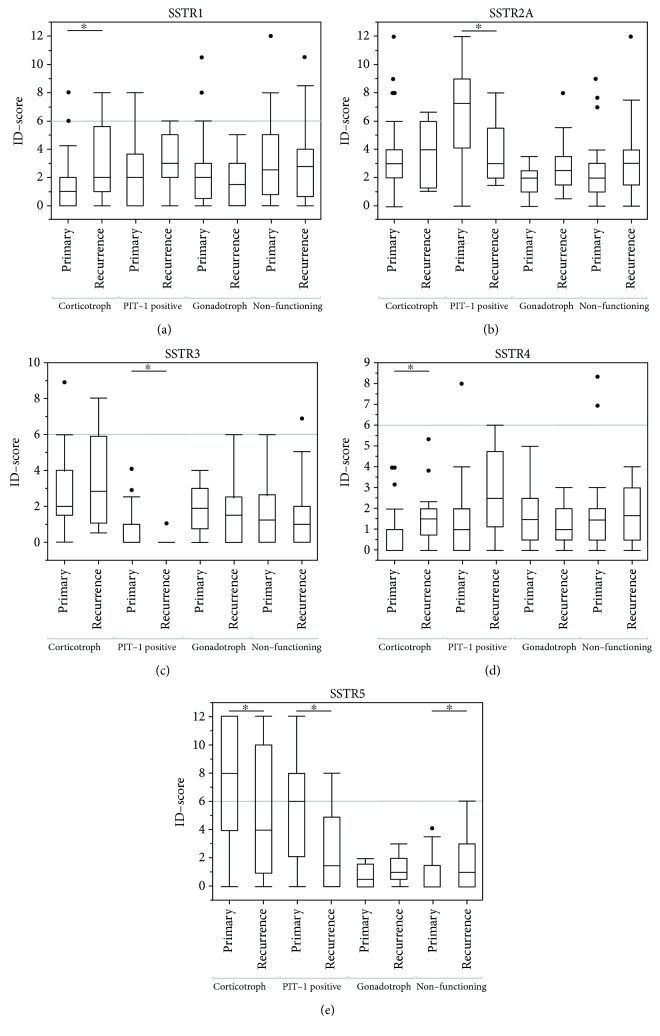
Differences of the expression of SSTR1 (a), SSTR2A (b), SSTR3 (c), SSTR4 (d), and SSTR5 (e) in primary and recurrent pituitary adenomas. Asterisks mark significant differences between primary and recurrent cases of an adenoma subgroup. Student's *t*-test with a significance level of *α* < 0.05 was applied. Outliers are represented as single points and were included in each analysis.

When comparing primary and recurrent adenomas, differences in the ID scores of some SSTRs were observed (Table 5). Recurrent CD cases showed a significantly higher expression of SSTR1 (*p* = 0.0351) and SSTR4 (*p* = 0.0174) while SSTR5 ID scores were lower than in primary corticotroph adenomas (*p* = 0.0370). SSTR5 expression was also lower in recurrent PIT-1-positive adenomas (*p* = 0.0350) but was higher in recurrent nonfunctioning adenomas (*p* = 0.0162) when compared to their specific primary subgroups. Additionally, SSTR2A and SSTR3 were lower in recurrent PIT-1-positive pituitary adenomas (*p* = 0.0049 and *p* = 0.0212, respectively). No significant differences in SSTR expression between primary and recurrent gonadotroph pituitary adenomas were observed (see Table 5 and Figures 4(a)–4(e)).

### 3.8. Anterior Pituitary Biopsies from CD Samples

Pituitary biopsies of 30 patients suffering from Cushing's disease did not reveal adenomatous tissue (30/148, 20%). For comparison with adenomatous tissue, we analyzed SSTR expression in these obtained samples as well. Twenty-two to twenty-four samples were suitable for expression analysis. The mean ID score of nonadenomatous samples was significantly higher for SSTR2A and SSTR5 (*p* = 0.0126 and 0.0008, respectively) than that of adenoma samples (see Supplementary Figure 1 and Table 1).

## 4. Discussion

### 4.1. SSTR Expression in CD

So far, only limited numbers of corticotroph pituitary adenomas have been investigated concerning the expression of different SSTRs with contradictory results.

In 1994, Greenman and Melmed analyzed SSTR 3, SSTR4, and SSTR5 in 33 pituitary adenomas and SSTR1 and SSTR2 in 27 pituitary adenomas using RT-PCR, including 2 and 3 ACTH-producing adenomas, respectively. While SSTR2 and SSTR4 were not detectable in corticotroph adenomas, one out of three cases showed expression of SSTR1 and one out of two for SSTR3 and SSTR5 [17, 18]. Another small group of corticotroph adenomas was analyzed by Miller et al. in 1995. RT-PCR and IHC of five corticotroph adenomas congruently showed no detection of SSTR3 and SSTR4 while mRNA and IHC levels for SSTR5 were high [19]. Contradictory to these results, mRNA of SSTR3 and SSTR4 was the only detectable somatostatin receptors via RT-PCR in one case of pituitary Cushing's disease in a cohort by Nielsen et al. in 2001. SSTR4 was not detected in any other of the 20 analyzed pituitary adenomas [20]. In 2006, Batista et al. performed quantitative RT-PCR and IHC for SSTR1-5 on thirteen pituitary adenomas. No mRNA for SSTR3 was detected while SSTR5-mRNA was found in all samples and also showed the highest IHC expression [22].

In this study, we analyzed the immunohistochemical expression of SSTR1-5 in the largest group of corticotroph adenomas so far. Immunohistochemical staining was chosen because of the possible application in neuropathology routine practice. In 88 cases, the expression was determined using an intensity distribution score, allowing a more precise quantitative description by including the signal intensity and the area of staining positivity [27]. This way we were able to show that SSTR5 expression was exceptionally high in corticotroph adenomas, while there were only a few samples that had low expression rates or none at all. The expression of SSTR2A and SSTR3 was also quite high but with a higher rate of low- or nonstainers. These receptors provide a promising target for medical treatment of ACTH-producing adenomas with somatostatin analogs. Furthermore, our thirty pituitary biopsy samples from patients undergoing surgery for CD with no adenoma detected during histopathological examination showed a significantly higher expression of SSTR2A and SSTR5. This confirms that the SSTR status in neoplastic tissue is altered compared to normal pituitary tissue.

### 4.2. SSTR as a Treatment Target in CD

The first substances engaging somatostatin receptors were mostly SSTR2 specific but did not show satisfactory efficacy in CD [14]. Later, it became evident that other SSTRs with higher expression rates, especially SSTR5, may be more efficient targets for somatostatin treatment [21]. Our data support this approach, since SSTR5 is highly expressed in CD. Over the past decades, more suitable substances with multireceptor-affinity have been developed. One of the newest is pasireotide. It shows a high affinity to SSTR1, 2, 3, and 5 [14] and has clinical efficacy [15, 16] with acceptable side effects [28, 29]. In light of the increased expression of SSTR1 in nonfunctioning adenomas, as presented in this study, pasireotide may also be a possible treatment option in this frequent adenoma subgroup.

In order to develop a medical treatment approach with long-lasting efficacy, it is also crucial to recognize the expression dynamics of somatostatin receptors that have been identified. A high receptor expression in the surgically resected tumor tissue prior to targeted treatment does not necessarily mean that targeting this receptor will generate the most and lasting clinical effect. For example, in vitro studies showed that the expression of SSTR2A is downregulated by applied glucocorticoids to human neuroendocrine cell lines [30]. In 2005, van der Hoek et al. demonstrated how dexamethasone decreases SSTR2A and 2B mRNA expression but not SSTR5 mRNA levels after 24 to 48 hours [31]. This may explain why SSTR2-targeting substances like octreotide did not show efficacy in CD patients with persistent or recurrent hypercortisolemia [14]. The findings described by van der Hoek et al. also indicate that SSTR5-specific substances may be less susceptible to this mechanism [31]. However, a phase III clinical trial by Colao et al., investigating pasireotide treatment in Cushing's disease, revealed that patients with exceptionally high urinary free cortisol levels were less likely to achieve normal cortisol levels with pasireotide [16]. Additionally, it has been suggested that USP-8 mutations predict the response to pasireotide in corticotroph adenomas [32].

These findings stress the importance of understanding alterations of hormone and receptor dynamics during treatment and the need for prospective correlation studies with immunohistochemistry. It needs to be clarified how the SSTR expression in corticotroph adenomas is influenced by somatostatin analogs and changes of cortisol levels, especially since an effective medical treatment would be applied for a longer period or even permanently. First studies focusing on pharmacokinetics and safety have been published [33, 34] but the receptor dynamics in Cushing's disease during somatostatin treatment are far from being completely understood.

### 4.3. SSTR in Recurrent Pituitary Adenomas

To get an impression on SSTR receptor dynamics, we took a closer look at expression levels of primary and recurrent PA. In CD, recurrent tumors had significantly higher expression rates of SSTR1 and SSTR4 and significantly lower expression of SSTR5. This is a finding not yet described in the literature. It is possible that prior treatment with somatostatins may have influenced expression levels by downregulation. Since commonly used substances have a high affinity to SSTR2A and SSTR5 [14], one would expect recurrent tumor tissue to have lower expression rates. However, this was observed for SSTR5 only. Most importantly, only a single patient received pasireotide prior to resection of a recurring corticotroph pituitary adenoma. In this case, the expression of all SSTRs was quite low including SSTR5. Unfortunately, we did not have access to the primary tumor tissue of this special case.

The observed difference cannot be explained sufficiently at the time. It shows that recurrent CD may not be as responsive to pasireotide as primary CD, which is a treatment option especially used in recurrent disease. To get a better insight into the dynamics of SSTR expression, it would be necessary to prospectively analyze a larger group of recurrent tumors that did receive prior somatostatin treatment.

Our study provides deeper insight into the SSTR expression variability in corticotroph adenoma tissue. We observed that SSTR5 is reduced in recurrent corticotroph adenomas indicating that their expression may be dynamically regulated. Therefore, the SSTR status should be gathered for each primary and recurrent sample during routine diagnostic in order to gain a better understanding of the receptor dynamics during treatment, which can only be partially mimicked in cell culture and animal models.

### 4.4. Limitations

Limitations of our study are the retrospective nature of the study and the nonstandardized treatment prior surgical resection that may have influenced the SSTR status of the tumors.

Although the tissue microarray method allows harmonized analysis of similar amounts of tumor tissue samples, the known shortcomings of this method are the main limitations of this study. In most cases, we took two cylinder samples of 1000 or 2000 *μ*m of representative areas, avoiding accumulation of stroma-rich, highly vascularized, or necrotic tissue. However, although histologically controlled, it is possible that the sample cylinders may have a vertical heterogeneity that could not be assessed beforehand. Since immunohistochemistry of SSTR1-5 is known to show homogenous staining in pituitary adenomas, this does probably not have a significant impact. We have utilized widely used SSTR antibodies [35–38].

The tissue samples were from a consecutive cohort of patients who were surgically treated from Cushing's disease in the authors' institution. The non-CD pituitary adenomas that were stained for comparative reasons were nonconsecutive cases from different TMA cohorts from previous research projects. Thus, the results of the non-CD cases may have a certain selection bias of tumors with sufficient tissue available. However, no particular selection was done by the authors. All available tissue samples on TMAs were stained and analyzed.

## 5. Conclusion

SSTR2A, SSTR3, and SSTR5 are highly expressed in corticotroph pituitary adenomas and differ from nonadenomatous tissue, providing a promising target for medical treatment with somatostatin analogs. Recurrent corticotroph pituitary adenomas show a different SSTR expression with lower SSTR5 levels and higher expression of SSTR1 and SSTR4. We proclaim the need to assess the expression of SSTR1-5 on a routine basis in corticotroph pituitary adenomas to allow individual treatment decisions and to gain experience about receptor dynamics during treatment.

## Figures and Tables

**Table 1:** Grading of immunohistopositivity according to Barresi et al. 2008.

Intensity distribution (ID) score = IS × ASP	0-12
*Immunostaining intensity (IS)*	
Negative	0
Weak	1
Moderate	2
Strong	3
*Area of staining positivity (ASP)*	
<5%	0
5–25%	1
26–50%	2
51–75%	3
76–100%	4

**Table 2:** Patient cohort characteristics.

	*n* (%)
Corticotroph	**88 (100)**
Gender	
Male	18 (20)
Female	70 (80)
Age at diagnosis	
Median (years)	49
Range (years)	16–79
Primary	68 (77)
Recurrence	20 (23)

PIT-1 positive	**55 (100)**
Gender	
Male	27 (49)
Female	28 (51)
Age at diagnosis	
Median (years)	50
Range (years)	17–75
Primary	47 (85)
Recurrence	8 (15)

Gonadotroph	**22 (100)**
Gender	
Male	11 (50)
Female	11 (50)
Age at diagnosis	
Median (years)	54
Range (years)	37–79
Primary	15 (68)
Recurrence	7 (32)

Nonfunctioning	**82 (100)**
Gender	
Male	45 (55)
Female	37 (45)
Age at diagnosis	
Median (years)	49
Range (years)	29-81
Primary	45 (55)
Recurrence	37 (45)

Nonadenomatous pituitary biopsy	**30 (100)**
Gender	
Male	8 (27)
Female	22 (73)
Age at diagnosis	
Median (years)	44
Range (years)	19-76
Primary	20 (33)
Recurrence	10 (67)

**Table 3:** Expression rates of SSTR1–5 in adenoma samples.

	*n* ^1^	Mean	95% CI	Negative (%)	C^2^	P^2^	G^2^	N^2^
*SSTR1*	236	2.47	2.15-2.78	65 (28)				
Corticotroph	79	1.79	1.34-2.25	23 (29)	—	0.0868	0.2141	0.001^∗^
PIT-1 positive	54	2.53	1.90-3.17	16 (30)	0.0868	—	0.9894	0.2089
Gonadotroph	22	2.52	1.32-3.72	6 (27)	0.2141	0.9894	—	0.3509
Nonfunctioning	81	3.07	2.46-3.68	20 (25)	0.001^∗^	0.2089	0.3509	—
*SSTR2A*	226	3.88	3.50-4.27	17 (8)				
Corticotroph	80	3.73	3.19-4.26	2 (3)	—	<.0001^∗^	0.0217^∗^	0.0126^∗^
PIT-1 positive	52	6.43	5.50-7.37	2 (4)	<.0001^∗^	—	<.0001^∗^	<.0001^∗^
Gonadotroph	18	2.19	1.31-3.08	3 (17)	0.0217^∗^	<.0001^∗^	—	0.4462
Nonfunctioning	76	2.70	2.21-3.19	10 (13)	0.0126^∗^	<.0001^∗^	0.4462	—
*SSTR3*	232	1.76	1.52-2.01	87 (38)				
Corticotroph	79	2.91	2.42-3.39	9 (11)	—	<.0001^∗^	0.0280^∗^	<.0001^∗^
PIT-1 positive	54	0.51	0.27-0.75	39 (72)	<.0001^∗^	—	0.0011^∗^	0.0026^∗^
Gonadotroph	20	1.98	1.23-2.73	5 (25)	0.0280^∗^	0.0011^∗^	—	0.1908
Nonfunctioning	78	1.42	1.06-1.78	34 (44)	<.0001^∗^	0.0026^∗^	0.1908	—
*SSTR4*	235	1.34	1.15-1.53	93 (40)				
Corticotroph	81	0.90	0.64-1.15	43 (53)	—	0.0238^∗^	0.0341^∗^	0.0013^∗^
PIT-1 positive	54	1.44	0.97-1.91	23 (43)	0.0238^∗^	—	0.5937	0.4281
Gonadotroph	22	1.64	1.02-2.25	6 (27)	0.0341^∗^	0.5937	—	0.9822
Nonfunctioning	78	1.64	1.31-1.98	21 (27)	0.0013^∗^	0.4281	0.9822	—
*SSTR5*	233	4.08	3.53-4.63	70 (30)				
Corticotroph	82	7.11	6.11-8.12	9 (11)	—	0.0003^∗^	<.0001^∗^	<.0001^∗^
PIT-1 positive	52	4.96	3.96-5.97	9 (17)	0.0003^∗^	—	<.0001^∗^	<.0001^∗^
Gonadotroph	21	0.90	0.50-1.31	12 (57)	<.0001^∗^	<.0001^∗^	—	0.7572
Nonfunctioning	78	1.16	0.83-1.49	41 (53)	<.0001^∗^	<.0001^∗^	0.7572	—

^1^The varying number of analyzed cases is based on the vertical heterogeneity of the tumor cylinders and in adequate fixation or staining of the samples in a few single cases. ^2^Corticotroph (C), PIT-1 positive (P), gonadotroph (G), and nonfunctioning (N) pituitary adenoma.

**Table 4:** Expression rates of SSTR1–5 regarding the ID score cut-off of 6.

	*n*	ID score ≥ 6 (%)	Pearson chi square test (ID score ≥ 6)
*SSTR1*	236	33 (14)	0.2599
Corticotroph	79	7 (9)	
PIT-1 positive	54	7 (13)	
Gonadotroph	22	3 (14)	
Nonfunctioning	81	16 (20)	
*SSTR2A*	226	58 (26)	<.0001^∗^
Corticotroph	80	19 (24)	
PIT-1 positive	52	33 (63)	
Gonadotroph	18	1 (6)	
Nonfunctioning	76	5 (7)	
*SSTR3*	232	18 (8)	<.0001^∗^
Corticotroph	79	15 (19)	
PIT-1 positive	54	0 (0)	
Gonadotroph	20	1 (5)	
Nonfunctioning	78	2 (3)	
*SSTR4*	235	4 (2)	0.3309
Corticotroph	81	0 (0)	
PIT-1 positive	54	2 (4)	
Gonadotroph	22	0 (0)	
Nonfunctioning	78	2 (3)	
*SSTR5*	233	74 (32)	<.0001^∗^
Corticotroph	82	48 (59)	
PIT-1 positive	52	25 (48)	
Gonadotroph	21	0 (0)	
Nonfunctioning	78	1 (1)	

**Table 5:** Expression differences between primary and secondary pituitary adenomas.

	Primary PA mean (*n*)	Recurrent PA mean (*n*)	*p* value
*Corticotroph*			
SSTR1	1.46 (62)	3.03 (17)	0.0351^∗^
SSTR2A	3.77 (63)	3.54 (17)	0.6985
SSTR3	2.78 (63)	3.43 (16)	0.3674
SSTR4	0.71 (65)	1.67 (16)	0.0174^∗^
SSTR5	7.68 (65)	4.95 (17)	0.0370^∗^
*PIT-1 positive*			
SSTR1	2.41 (46)	3.25 (8)	0.3047
SSTR2A	6.93 (44)	3.69 (8)	0.0049^∗^
SSTR3	0.58 (46)	0.13 (8)	0.0212^∗^
SSTR4	1.20 (46)	2.81 (8)	0.0673
SSTR5	5.40 (44)	2.56 (8)	0.0350^∗^
*Gonadotroph*			
SSTR1	2.83 (15)	1.86 (7)	0.3551
SSTR2A	1.68 (11)	3.00 (7)	0.2130
SSTR3	2.04 (13)	1.86 (7)	0.8391
SSTR4	1.80 (15)	1.29 (7)	0.3606
SSTR5	0.75 (14)	1.21 (7)	0.3232
*Nonfunctioning*			
SSTR1	3.08 (45)	3.06 (36)	0.9715
SSTR2A	2.40 (42)	3.07 (34)	0.1853
SSTR3	1.61 (42)	1.20 (36)	0.2581
SSTR4	1.53 (43)	1.78 (35)	0.4520
SSTR5	0.79 (43)	1.61 (35)	0.0162^∗^

## Data Availability

The data used to support the findings of this study are available from the corresponding author upon request.

## Supplementary Materials

Supplementary Materials Supplementary Figure 1: expression rates of SSTR1–5 in nonadenomatous tissue samples. Supplementary Table 1: SSTR2A and SSTR5 in nonadenomatous pituitary tissue. Asterisks mark significant differences between using Student's *t*-test with a significance level of *α* < 0.05. Supplementary Table 2: expression of SSTR1–5 in pituitary adenomas according to the previous WHO classification.
