# Supplementary material for: High Expression of Somatostatin Receptors 2A, 3, and 5 in Corticotroph Pituitary Adenoma
Source: Int J Endocrinol. 2018 Dec 9;2018:1763735. doi: 10.1155/2018/1763735 (PMC6304820; doi:10.1155/2018/1763735)
Supplement: Supplementary Materials — Supplementary Figure 1: expression rates of SSTR1–5 in nonadenomatous tissue samples. Supplementary Table 1: SSTR2A and SSTR5 in nonadenomatous pituitary tissue. Asterisks mark significant differences between using Student's t-test with a significance level of α < 0.05. Supplementary Table 2: expression of SSTR1–5 in pituitary adenomas according to the previous WHO classification. [file 1763735.f1.docx]

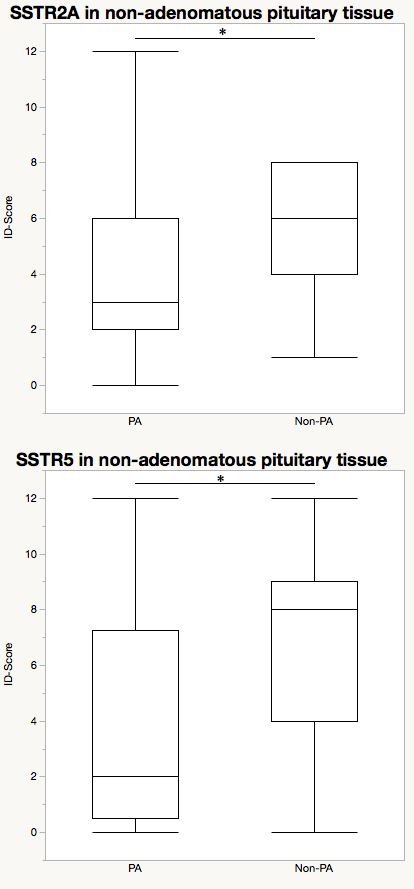


**Supplementary Figure 1**: SSTR2A and SSTR5 in non-adenomatous pituitary tissue. Asterisks mark significant differences between using the Student’s t-test with a significance level of α < 0.05.

Supplementary Table 1: Expression rates of SSTR1 – 5 in non-adenomatous tissue samples.

|  | **Pituitary adenomas**  **mean (n)** | **Non-adenomatous pituitary samples**  **mean (n)** | **p-value** |
| --- | --- | --- | --- |
| SSTR1 | 2.47 (236) | 2.42 (24) | 0.9032 |
| SSTR2A | 3.88 (226) | 5.25 (24) | 0.0126* |
| SSTR3 | 1.76 (231) | 2.77 (22) | 0.0528 |
| SSTR4 | 1.34 (235) | 2.17 (23) | 0.0806 |
| SSTR5 | 4.08 (233) | 6.78 (23) | 0.0008* |

Supplementary Table 2: Expression of SSTR1 – 5 in pituitary adenoma according to the previous WHO classification.

|  | n  (%) | Positive  (%) | Negative  (%) | Mean | ID score >/= 6 (%) |
| --- | --- | --- | --- | --- | --- |
| **SSTR1** | 263 (100) | 189 (72) | 74 (28) | 2.40 | 33 (13) |
| CD | 99 (100) | 70 (71) | 29 (29) | 1.75 | 7 (7) |
| Inactive PA | 102 (100) | 76 (75) | 26 (25) | 2.99 | 19 (19) |
| Acromegaly | 40 (100) | 26 (65) | 14 (35) | 2.48 | 6 (15) |
| Prolactinoma | 18 (100) | 14 (78) | 4 (22) | 2.40 | 1 (6) |
| Thyrotropinoma | 4 (100) | 3 (75) | 1 (25) | 3.00 | 0 (0) |
| **SSTR2A** | 254 (100) | 236 (93) | 18 (7) | 3.93 | 67 (26) |
| CD | 101 (100) | 98 (97) | 3 (3) | 3.68 | 24 (24) |
| Inactive PA | 93 (100) | 80 (86) | 13 (14) | 2.58 | 6 (7) |
| Acromegaly | 39 (100) | 37 (95) | 2 (5) | 6.79 | 27 (69) |
| Prolactinoma | 17 (100) | 17 (100) | 0 (0) | 4.38 | 6 (35) |
| Thyrotropinoma | 4 (100) | 4 (100) | 0 (0) | 9.75 | 4 (100) |
| **SSTR3** | 258 (100) | 161 (62) | 97 (38) | 1.78 | 22 (9) |
| CD | 99 (100) | 85 (86) | 14 (14) | 2.80 | 19 (19) |
| Inactive PA | 97 (100) | 59 (61) | 38 (39) | 1.54 | 3 (3) |
| Acromegaly | 40 (100) | 12 (30) | 28 (70) | 0.60 | 0 (0) |
| Prolactinoma | 18 (100) | 4 (22) | 14 (78) | 0.37 | 0 (0) |
| Thyrotropinoma | 4 (100) | 1 (25) | 3 (75) | 0.25 | 0 (0) |
| **SSTR4** | 261 (100) | 159 (61) | 102 (39) | 1.35 | 4 (2) |
| CD | 99 (100) | 48 (48) | 51 (52) | 0.99 | 0 (0) |
| Inactive PA | 100 (100) | 73 (73) | 27 (27) | 1.64 | 2 (2) |
| Acromegaly | 40 (100) | 23 (58 | 17 (42) | 1.14 | 0 (0) |
| Prolactinoma | 18 (100) | 13 (72) | 5 (28) | 2.18 | 2 (11) |
| Thyrotropinoma | 4 (100) | 2 (50) | 2 (50) | 1.38 | 0 (0) |
| **SSTR5** | 262 (100) | 189 (72) | 73 (28) | 4.30 | 91 (35) |
| CD | 104 (100) | 94 (90) | 10 (10) | 6.96 | 61 (59) |
| Inactive PA | 98 (100) | 46 (47) | 52 (53) | 1.08 | 1 (1) |
| Acromegaly | 38 (100) | 35 (92) | 3 (8) | 5.84 | 23 (61) |
| Prolactinoma | 18 (100) | 10 (55) | 8 (45) | 2.25 | 3 (17) |
| Thyrotropinoma | 4 (100) | 4 (100) | 0 (0) | 8.38 | 3 (75) |
